# Adrenal Ganglioneuroma With Radiology-Pathology Correlation

**DOI:** 10.7759/cureus.69584

**Published:** 2024-09-17

**Authors:** Alvaro Rueda-de-Eusebio, Montserrat de la Torre Serrano, Alejandro Victoria Artalejo, Ramiro Mendez

**Affiliations:** 1 Department of Radiology, Hospital Clínico San Carlos, Madrid, ESP; 2 Department of Pathology, Hospital Clínico San Carlos, Madrid, ESP; 3 Department of Radiology, Hospital Universitario Rey Juan Carlos, Móstoles, ESP

**Keywords:** adrenal ganglioneuroma, adrenal glands, adrenal incidentalomas, ct, mri

## Abstract

This report presents the case of a 62-year-old male with an incidentally discovered adrenal mass, ultimately diagnosed as a ganglioneuroma after laparoscopic adrenalectomy. Imaging findings, of a 9 cm lobulated mass with heterogeneous enhancement on MRI, were unspecific and malignancy could not be excluded. Histological examination of the specimen revealed a well-demarcated tumor composed of Schwann cells and ganglion cells, confirming the diagnosis. Postoperative follow-up showed no recurrence. This case highlights the critical role of histopathological evaluation for definitive diagnosis, as imaging alone may be insufficient to distinguish adrenal ganglioneuroma from other potential malignancies due to its nonspecific radiological features.

## Introduction

Ganglioneuromas are uncommon tumors that develop from neural crest cells, with adrenal ganglioneuromas representing around 20% of all cases. The adrenal glands, small structures located deep within the retroperitoneum, are frequently affected by various conditions. In recent years, improvements in cross-sectional imaging techniques have resulted in an increase in the incidental discovery of adrenal masses during abdominal scans performed for other unrelated purposes. Ganglioneuromas make up roughly 0.3-2% of these adrenal incidentalomas [1-3].

These tumors are well-differentiated, consisting entirely of ganglion cells and Schwannian stroma, and unlike other peripheral neural tumors such as neuroblastomas or ganglioneuroblastomas, they lack neuroblasts, intermediate cells, or mitotic figures. Due to their incidental discovery, the true prevalence and incidence of adrenal ganglioneuromas are largely unknown, with most of the available data coming from case reports and series [1,4].

Diagnosing adrenal ganglioneuromas is particularly challenging, as they tend to be large tumors. Imaging features on non-contrast CT scans often reveal densities greater than 10 HU, while contrast-enhanced washout is typically below 50%. Other findings include calcifications, heterogeneity, hyperintensity on T2-weighted MRI images, and potential increased uptake on 18F-FDG-PET/CT. However, these characteristics are non-specific and can overlap with other adrenal tumors, making it difficult to rule out malignancy. As a result, adrenal ganglioneuroma is rarely suspected before surgery [4].

In this context, this report presents a case of adrenal incidentaloma from our institution that is very representative of the path these patients usually follow until a definitive diagnosis is reached.

## Case presentation

A 62-year-old male was referred to the Pneumology Department due to a positive tuberculin test. A CT scan of the chest and abdomen was performed, in which no findings related to tuberculosis were identified, but a left adrenal mass with a maximum diameter of 9 cm was detected.

Clinically, the patient was asymptomatic. He had a history of high blood pressure, well controlled with a standard dose of enalapril. He did not present digestive tract symptoms such as nausea or diarrhea. The physical exam was unremarkable. Various laboratory tests were performed, including a comprehensive blood analysis, with hematological and biochemical profiles, with all values in range. A 24-hour urine sample was taken, where metanephrines were normal. Basal blood cortisol was 13.5 μg/dL (5-25), nocturnal cortisol was 1.3 μg/dL (normal if >50% reduction), and blood DHEA-s was 1.4 μmol/L (0.62-6.62), all in the normal range.

**Figure 1:**
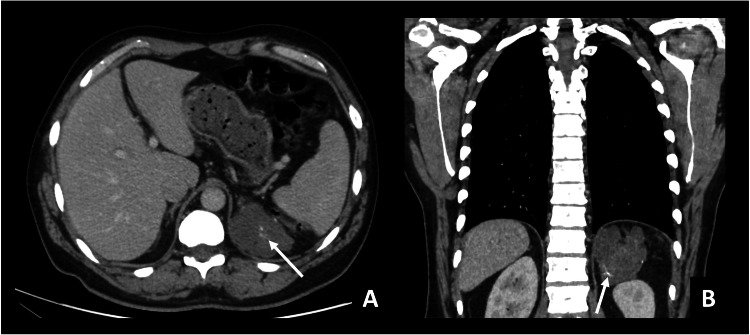
Chest CT with IV iodinated contrast (venous phase) Axial (A) and coronal (B) planes. Polylobulated mass in the left adrenal gland. It is homogeneous, with relatively low attenuation/mild enhancement (30 HU in portal venous phase). There are small calcification foci within the mass (arrows).

The CT with IV iodinated contrast (Figure 1) showed a mass on the left adrenal gland, measuring 9 cm. It presented relatively homogeneous low attenuation (30 HU) with small calcium foci. Having no specific criteria for the diagnosis of a benign adrenal mass (adenoma or myelolipoma), an abdominal MRI was recommended.

**Figure 2:**
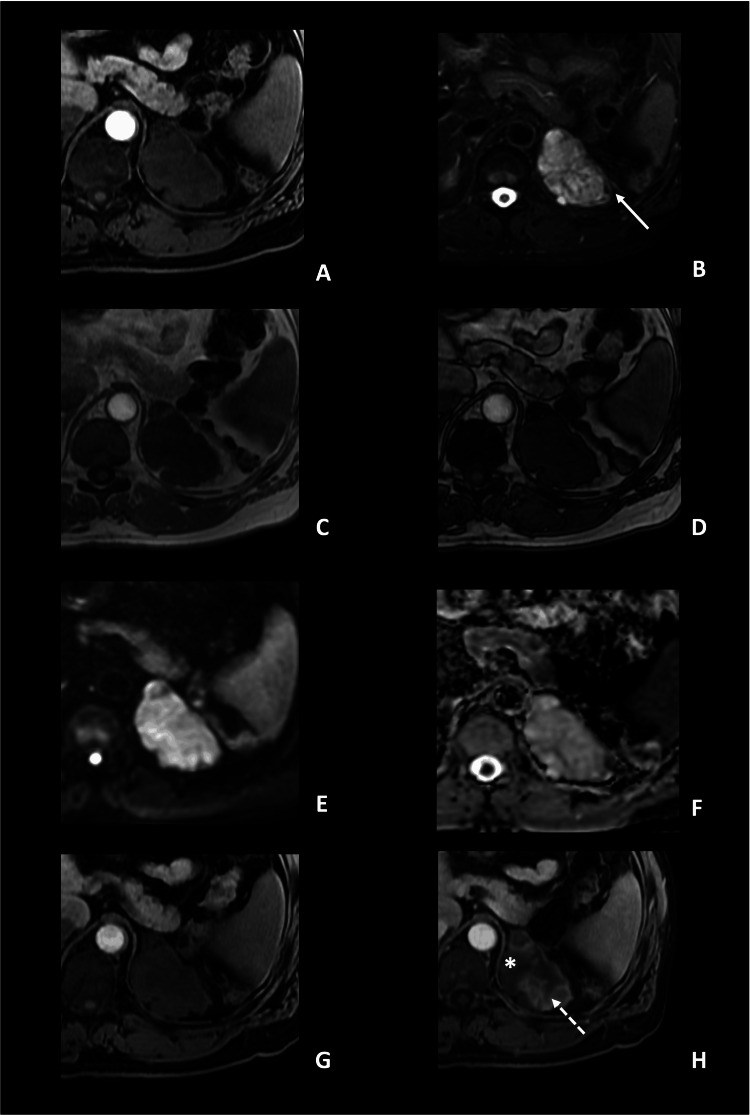
Abdominal MRI (3T), axial planes. Axial planes. A: T1WI; B: T2WI with fat suppression; C, D: chemical shift sequences with images in-phase (C) and out-of-phase (D); E: diffusion-weighted images, with b-value of 800 (E) and ADC map (F) and sequence after gadolinium contrast injection (G: early phase, H: delayed phase). Left adrenal mass with homogeneous low SI on T1WI and heterogeneous high SI on T2WI. In the lateral aspect of the mass, there are some low SI foci on T2WI (arrow), which correspond with calcifications, visible on CT. In the chemical shift sequence, there is no evidence of intravoxel microscopic fat, and on the DWI, no diffusion restriction was present (ADC value of 2.1 x 10-3 mm^2^/s). In the delayed phase of the dynamic post-contrast sequences (H), there is a heterogeneous enhancement, with areas of delayed enhancement (dashed arrow) and non-enhancing areas (*).

In the MRI (Figure 2), the left adrenal mass presented lobulated margins and measured 9 x 5.5 x 6.3 cm (CC x T x AP). It showed low signal intensity (SI) on T1-weighted images, and heterogeneous high signal intensity on T2-weighted sequences, with no restricted diffusion. Chemical shift sequences did not show intravoxel fat content. In the dynamic postcontrast sequence, the lesion did not present avid enhancement. In fact, enhancement was heterogeneous, having areas of progressive delayed enhancement and non-enhancing areas. Again, the lesion did not meet the criteria for adenoma or myelolipoma and did not present typical radiologic features of other common tumors such as pheochromocytoma, usually showing more contrast enhancement in earlier phases. We also could not rule out malignant lesions such as adrenocortical carcinoma.

The results were discussed with the Endocrinology Department, the Endocrine Surgery Department, and the patient himself. A histological confirmation was needed and the final agreement was that a thorough histologic study of the whole adrenal mass was preferred over a needle biopsy. Consequently, a laparoscopic adrenalectomy was performed, without any perioperative complications or incidents, and the patient was discharged four days after surgery.

**Figure 3:**
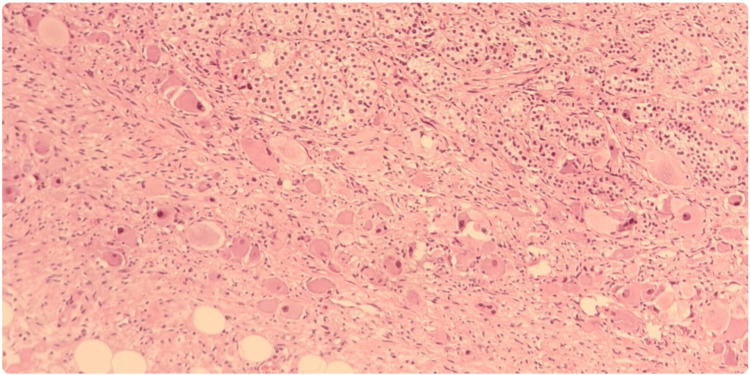
Histologic analysis of the surgical specimen. Hematoxylin-eosin 200x: In the upper right margin, the fasciculated layer of the adrenal cortex is identified, composed of cells with clear cytoplasm and small lipid vacuoles giving a foamy appearance. Interspersed with them, Schwann spindle cells and ganglion cells are identified.

**Figure 4:**
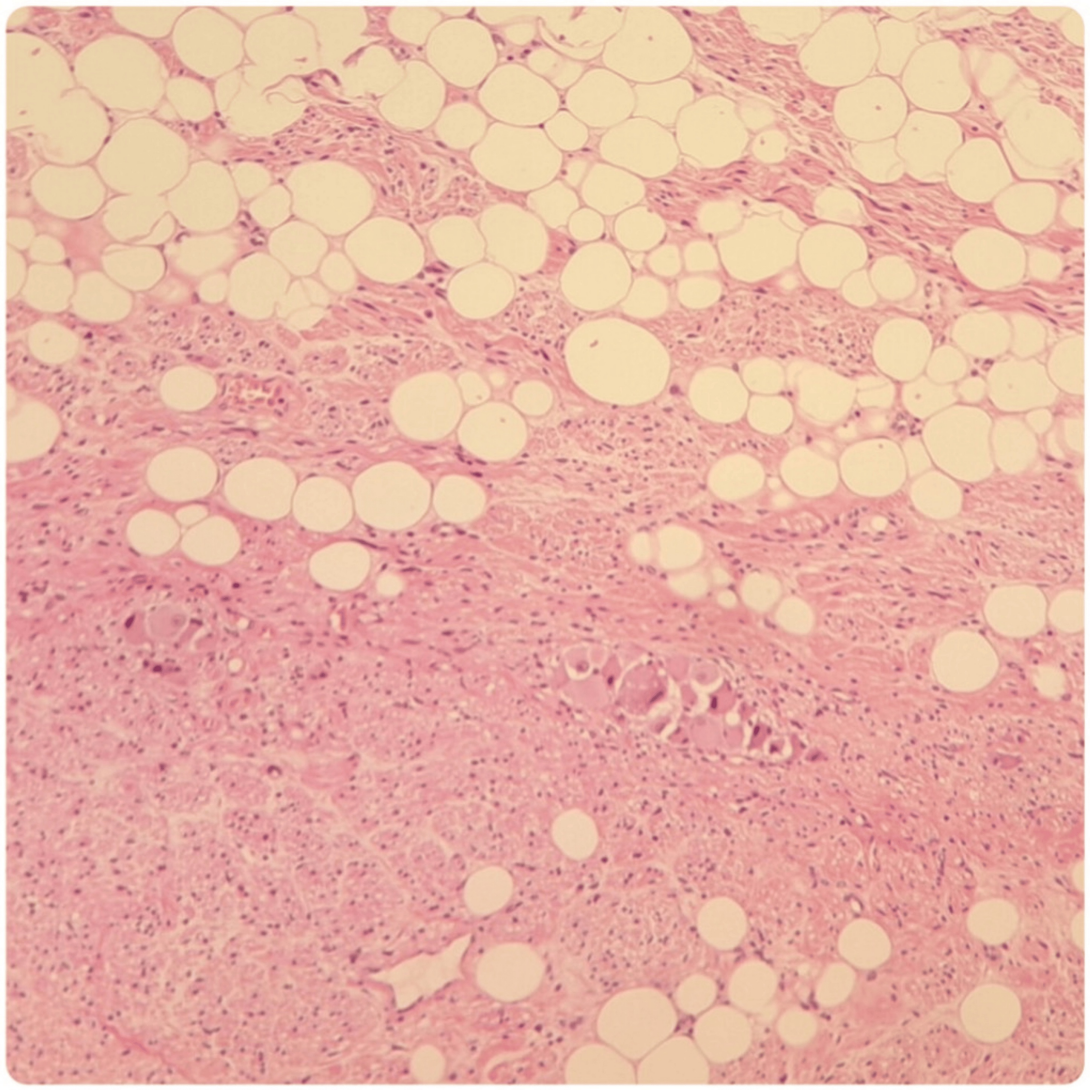
Histologic analysis of the surgical specimen. Hematoxylin-eosin 100X; A mixture of Schwann cells, mature ganglion cells, and adipose tissue is identified. C: Hematoxylin-eosin 400X; Ganglion cells that show compact eosinophilic cytoplasm with well-defined cell borders, a single eccentric nucleus, and a prominent nucleolus are shown.

**Figure 5:**
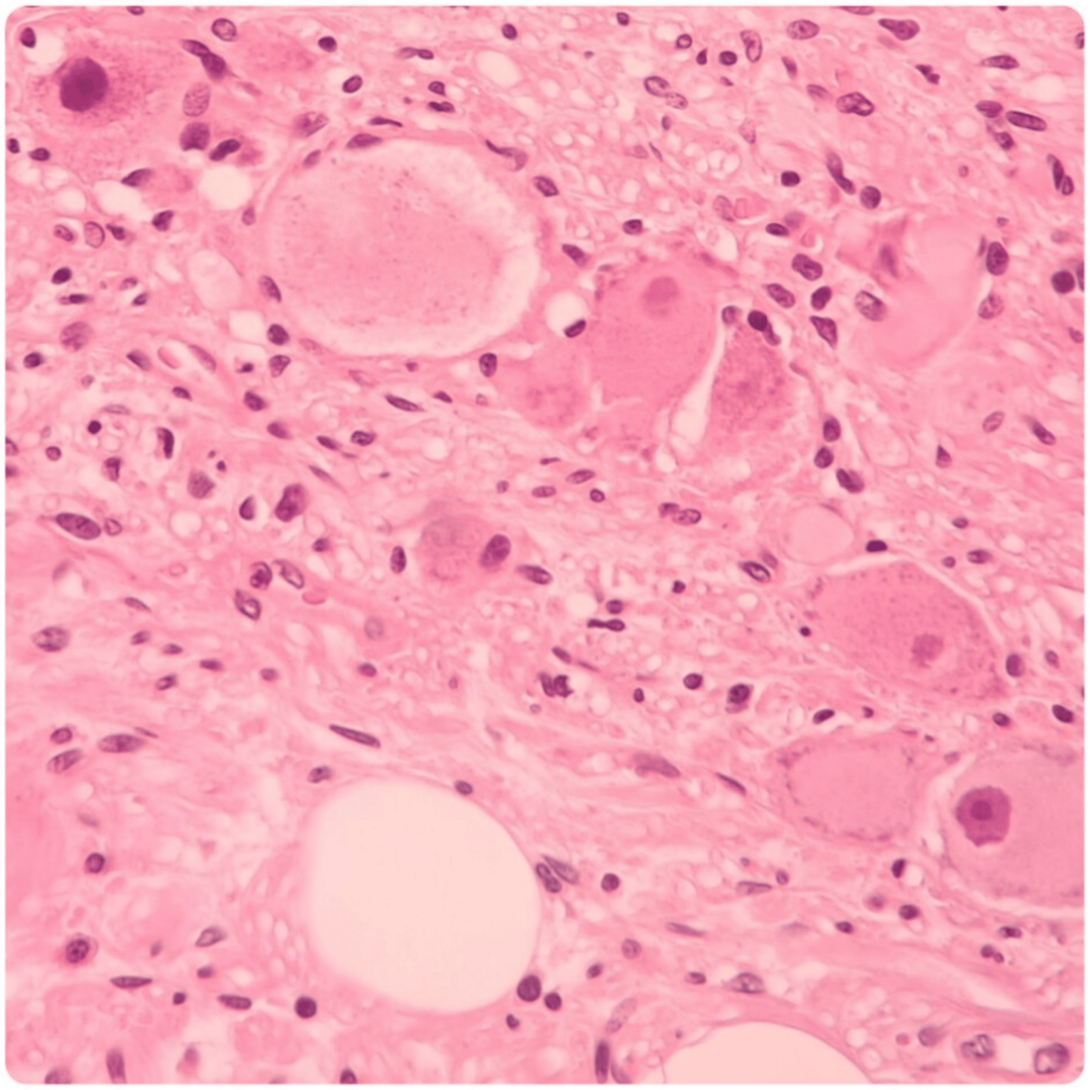
Histologic analysis of the surgical specimen. Hematoxylin-eosin 400x: Ganglion cells that shows compact eosinophilic cytoplasm with well-defined cell borders, single eccentric nucleus, and a prominent nucleolus are shown.

The macroscopic study of the surgical specimen showed a white, solid, and homogeneous surface. In the microscopic analysis (Figures 3-5), the lesion was composed of a mixture of Schwann cells, mature ganglion cells, and adipose tissue. The lesion had a well-demarcated border, and the surgical margin was not affected. There was no evidence of mitotic activity, necrosis, or atypia. All those features were consistent with an adrenal ganglioneuroma.

One year after the procedure, a follow-up CT scan was performed, in which no tumor relapse was identified. Since then, the patient has been undergoing regular clinical and analytical follow-ups, with no further incidents.

## Discussion

Ganglioneuromas are uncommon, well-differentiated tumors that originate from neural crest cells. They are most often found in the posterior mediastinum (41.5% of cases), retroperitoneum (37.5%), adrenal glands (21%), and neck (8%) [1,3]. However, they can also appear in rare locations such as the spermatic cord, heart, bones, and intestines [1]. Adrenal ganglioneuromas tend to occur more frequently in people in their 40s and 50s, whereas retroperitoneal and mediastinal ganglioneuromas are more commonly observed in children and young adults. The average tumor size reported in studies is 8 cm, with thoracic ganglioneuromas generally being larger than non-thoracic ones at diagnosis. Some research indicates a slight female predominance, though others suggest equal incidence across genders. A familial predisposition has been proposed, along with potential associations with Turner syndrome and multiple endocrine neoplasia type II [1,3-5].

Adrenal ganglioneuromas are typically asymptomatic and hormonally silent, even when they grow to significant sizes. Occasionally, they may cause abdominal pain due to a mass effect. Up to 30% of patients with ganglioneuroma may exhibit elevated plasma and urinary catecholamine levels, although they often lack symptoms of catecholamine excess. Rarely, these tumors can secrete vasoactive intestinal peptides or steroid hormones, such as cortisol or testosterone [1,4,6].

Imaging features

Adrenal ganglioneuromas are challenging to identify on conventional radiography, although in some cases, when reaching a large size, the semiology of a retroperitoneal mass can be appreciated, with effacement and displacement of retroperitoneal interfaces. Ultrasound (US) often reveals a homogeneous, hypoechoic mass with well-defined borders. On CT, they typically appear as well-defined, oval, crescentic, or lobulated masses, sometimes with a thin capsule. They display low, homogeneous attenuation on unenhanced CT scans and show slight to moderate enhancement after contrast, which can be homogeneous or heterogeneous. Calcifications, seen in 20-69% of cases, are a key indicator of ganglioneuromas. On MRI, these tumors exhibit low signal intensity on T1-weighted images and heterogeneous high signal intensity on T2-weighted images, attributed to the presence of myxoid stroma and relatively few ganglion cells. A whorled appearance on T2 images is characteristic due to the interlacing bundles of Schwann cells and collagen fibers. Gadolinium enhancement varies, with these tumors typically not enhancing early but accumulating contrast material in delayed phases. Ganglioneuromas can also show uptake on MIBG (131-metaiodobenzylguanidine) scans, particularly in association with catecholamine production. On FDG-PET, a standardized uptake value (SUV) of 3.0 or higher may differentiate malignant from benign adrenal lesions, although the evidence remains limited [2,3,7-11].

The imaging findings for adrenal ganglioneuromas are not definitive, with a preoperative misdiagnosis rate of 64.7% [8]. A well-defined adrenal mass featuring punctate calcifications, a density ranging from 10 to 60 HU on non-contrast CT, along with hypo-intense T1 and hyper-intense T2 signals on MRI, may indicate a ganglioneuroma, but other adrenal tumors could present similarly. The differential diagnosis includes adrenal adenomas, adrenocortical carcinomas, pheochromocytomas, and rare tumors like extramedullary plasmacytoma, primary lymphoma, or Schwannoma. These tumors may also present as composite or collision tumors, further complicating preoperative diagnosis, which can only be confirmed histologically after a thorough examination of the complete surgical specimen [3,4,12].

Pathology

Histologically, ganglioneuromas consist of ganglion cells and Schwannian stroma. By definition, they lack neuroblasts or intermediate cells, distinguishing them from neuroblastomas or ganglioneuroblastomas. Gross pathology typically reveals a firm, well-circumscribed tumor, which may appear trabeculated or whorled and range in color from white to yellow. Ganglioneuromas do not usually have a true capsule. Immunohistochemically, these tumors are positive for markers such as S-100, vimentin, synaptophysin, and neuron-specific enolase [1,3,11,13].

Treatment

Complete surgical resection is the preferred treatment for adrenal ganglioneuromas, as it allows for thorough sampling of the tumor, which is essential for an accurate and definitive diagnosis. Preoperative biopsies are often debated due to the risk of misdiagnosis, tumor dissemination, and the possibility of composite tumors. Given the excellent prognosis of adrenal ganglioneuromas, some argue that a definitive preoperative diagnosis could avoid surgery. However, imaging alone cannot exclude malignant tumors like adrenocortical carcinomas, and biopsy complications may outweigh the benefits. Therefore, histologic confirmation remains essential [4,14,15].

Adrenalectomy is usually the surgery of choice, although the tumor’s hormonal activity, location, and relationship to nearby structures may influence the approach. Adjuvant therapy is not required, as adrenal ganglioneuromas generally have an excellent prognosis [8,16,17].

Prognosis

Ganglioneuromas fall into the favourable histology category according to the International Neuroblastoma Pathology Committee. Although this classification is based on pediatric populations, evidence suggests it may also apply to adults. Surgical removal ensures a complete diagnosis, and one post-operative CT scan is typically recommended to check for residual tumor tissue. A 2021 review of 201 cases of surgically removed adrenal ganglioneuromas found no recurrences. In rare cases, metastatic ganglioneuromas may represent metastases from neuroblastomas or ganglioneuroblastomas that have matured, and these patients have excellent outcomes. Malignant transformation of a ganglioneuroma into a neuroblastoma or ganglioneuroblastoma is highly unlikely, as mature ganglion cells lack the capacity for such dedifferentiation and proliferation [1,4].

## Conclusions

Adrenal ganglioneuromas are rare, well-differentiated tumors arising from neural crest cells. These lesions are typically discovered incidentally and are usually non-functioning, so they do not produce hormones. While adrenalectomy remains the gold standard for treating adrenal ganglioneuromas, preoperative differential diagnosis can be particularly challenging. Ultimately, histological analysis is required to confirm this uncommon diagnosis.
